# Preoperative serum CA19-9 predicts postoperative pancreatic fistula in PDAC patients: retrospective analysis at a single institution

**DOI:** 10.1186/s12893-022-01825-3

**Published:** 2022-10-28

**Authors:** Hiroki Sugita, Hirohisa Okabe, Daisuke Ogawa, Hiroki Hirao, Daisuke Kuroda, Katsunobu Taki, Shinjiro Tomiyasu, Masahiko Hirota

**Affiliations:** Department of Surgery, Kumamoto Regional Medical Center, 5-16-10 Honjo, Chuo-Ku, Kumamoto, 860-0811 Japan

**Keywords:** Body mass index, Postoperative complication, Postoperative pancreatic fistula, CA19-9, Long-term outcome

## Abstract

**Background:**

Postoperative pancreatic fistula (POPF) is a critical complication of pancreatectomy in patients with pancreatic ductal adenocarcinoma (PDAC). Recent papers reported that serum carbohydrate antigen (CA)19-9 levels predicted long-term prognosis. We investigated whether preoperative serum CA19-9 levels were associated with POPF in PDAC patients.

**Methods:**

This cohort study was conducted at a single institution retrospectively. Clinicopathologic features were determined using medical records.

**Results:**

Among of 196 consecutive patients who underwent pancreatectomy against PDAC, 180 patients whose CA19-9 levels were above the measurement sensitivity, were registered in this study. The patients consisted of 122 patients who underwent pancreaticoduodenectomy and 58 patients who underwent distal pancreatectomy. Several clinicopathological factors, including CA 19-9 level, as well as surgical factors were determined retrospectively based on the medical records. Patients with high CA19-9 levels had a significantly higher incidence of POPF than those with low levels (43.9 vs. 13.0%, P < 0.0001). The receiver operating characteristic curves calculated that the cutoff CA19-9 value to predict POPF was 428 U/mL. CA19-9, BMI, curability, and histology were statistically significant risk factors for POPF by univariate analysis. Multivariate analysis showed that CA19-9 and BMI levels were statistically significant independent risk factors for POPF. CA19-9 levels were correlated with both histology and curability. Disease free survival and overall survival of patients with higher levels of CA19-9 were significantly shorter than that of patients with lower levels of preoperative serum CA19-9.

**Conclusions:**

In patients undergoing pancreatectomy for PDAC, higher preoperative CA19-9 levels are a significant predictor for POPF.

**Supplementary Information:**

The online version contains supplementary material available at 10.1186/s12893-022-01825-3.

## Introduction

Pancreatic ductal adenocarcinoma (PDAC) is prone to recurrence and the long-term prognosis is poor [1–3]. Pancreatectomy is the most effective treatment available for resectable PDAC. Pancreaticoduodenectomy (PD) or distal pancreatectomy (DP) is selected by tumor location. Papers reported that the morbidity and mortality after pancreaticoduodenectomy were 20–50% and 1–8%, respectively [4–6]. In contrast to this, the morbidity and mortality after distal pancreatectomy were 25–64% and 0–3%, respectively [7]. Pancreatic fistula (PF) as a postoperative complication occurred in 12–27% of the patients who underwent PD, in 16–51% of the patients who underwent DP and sometimes led to severe conditions in patients [7–11].

The effects of adjuvant chemotherapy on long-term prognosis of PDAC have been confirmed by several randomized control studies [2, 12, 13]. However, it is difficult to perform immediate adjuvant chemotherapy in the cases with post-operative pancreatic fistula (POPF). Therefore, recognizing the risk for POPF before pancreatectomy is critical.

Serum carbohydrate antigen (CA)19–9 levels are elevated in 69–92% of PDAC patients [14]. Papers had reported that elevated preoperative CA19-9 levels predicted long-term prognosis [15, 16]. On the other hand, there is no report that adequately examine the relationship between preoperative CA 19–9 levels and postoperative complication in PDAC patients. We investigated whether preoperative CA19-9 levels were associated with postoperative complications such as POPF.

### Patients and methods

This study was conducted at Kumamoto Regional Medical Center, Kumamoto, Japan. This study has been approved by the institutional ethics committee (#19-019). The informed consent was obtained from all enrollees. The cohort consisted of consecutive 196 patients who underwent PD or DP for PDAC between December 2002 and August 2020. The clinicopathological findings of 180 patients whose preoperative CA19-9 levels were more than measurement sensitivity, were determined retrospectively based on the medical record. Cancer staging was performed according to the International Union Against Cancer (UICC, 8th edition) classification. Residual cancer status was defined using the standard R-classification (R0, R1, and R2). Histological classification was determined using international classification of diseases for oncology (ICD-O) code. PF was determined using the International Study Group of Pancreatic Fistula (ISGPF) 2005 classification, and grade B and C were defined as PF [6, 9]. Post-operative complication was determined by Clavien Dindo criteria and grade 2 and over was defined as post-operative complication [17]. The International Study Group of Pancreatic Surgery (ISGPS) classification was used to define delayed gastric emptying (DGE), and grade B and C were defined as DGE [18]. Operative risk was determined using the American Society of Anesthesiologists Physical Status classification system (ASA-PS) [19].

Several clinicopathological factors such as gender, age, body mass index (BMI), ASA-PS, histological types, tumor size, tumor factor (T1-T4), lymph node metastases, pathological stage, neoadjuvant chemotherapy, size of Wirsung duct, total bilirubin levels and CA19-9 levels were retrospectively determined as the risk factors for POPF, along with surgical factors such as operation time, amount of bleeding, and curability (R 0 / R1/ R2) based on the medical record. Surgical site infection, length of hospital stay, hospital mortality and adjuvant chemotherapy also were determined. Furthermore, long-term postoperative follow-up and prognostic studies were conducted.

### Statistical analysis

Data were analyzed using JMP® 15.0 (SAS Institute Inc., Cary, NC, USA). The Wilcoxon and Kruskal–Wallis tests were used to compare between-group differences in the continuous variables. Proportions were analyzed with the Chi-squared test. Risk factors with p-values < 0.05 on univariate analysis were subjected to multivariate logistic regression. Disease free survival (DFS) and overall survival (OS) curves were plotted using the Kaplan–Meier method, and these comparisons were made using the log-rank test. All p-values less than 0.05 were considered statistically significant.

## Results

Among of one hundred ninety-six patients undergoing pancreatectomy for PDAC, 16 patients were excluded from the analysis, because their CA19-9 levels were below the measurement sensitivity threshold (< 1 U/mL). Finally, 180 patients, including 122 patients undergoing PD and 58 patients who underwent DP for PDAC, were registered. Clinicopathologic features in the cohort of 180 patients (96 men, 84 women; mean age, 68.9 ± 0.7 years) are summarized in Table 1. Median CA19-9 was 148 U/mLand; mean BMI was 22.0; mean tumor size was 3.34 cm.; the T-factor with the highest number of patients is T3 (n = 165, 91.7%); the most numerous cancer stage was IIB (n = 77, 42.8%). Portal vein resection was performed in 50 of the 122 patients who underwent PD and 3 of the 58 patients who underwent DP. In patients who underwent PD, duct-to-mucosa pancreaticojejunostomy (PJ), end-to -side invaginating pancreaticojejunostomy and duct-to-mucosa pancreaticogastrostomy (PG) as pancreatic anastomosis, were performed following PD. Duct-to-mucosa PJ was performed on 75 patients, end-to-side invaginating PJ on 36 patients, and duct-to-mucosa PG on 11 patients. Average annual number of operators was 3.84. A total of 18 surgeons have performed pancreatectomies at the hospital from December 2002 to August 2020.

**Table 1:** Clinicopathologic features in the cohort of 180 patients enrolled in this study

Variables	
Gender (male/female)	96/84
Age	68.9 ± 0.7
BMI	22.0 ± 0.2
ASA-PS	
1	40 (22.2%)
≥ 2	140 (77.8%)
Histology: well-differentiated	72 (40%)
Mean CA19-9 (U/mL)	1065 ± 319
Median CA 19–9 (U/mL)	148
Tumor size (cm)	3.34 ± 0.11
pT(UICC)	
1	5 (2.8%)
2	5 (2.8%)
3	165 (91.7%)
4	5 (2.8%)
pN(UICC)	
0	57 (31.7%)
1	78 (43.3%)
2	45 (25.0%)
pStage(UICC)	
IA	5 (2.8%)
IB	3 (1.7%)
IIA	50 (27.8%)
IIB	77 (42.8%)
III	45 (25.0%)
R0	145 (80.6%)
Surgery	
PD	122
DP	58
Neoadjuvant chemotherapy	28 (15.6%)
Postoperative complications	83 (46.1%)

### Short-term outcomes of pancreatectomy for PDAC

Surgery-related death occurred in 3 patients (1.7%); the cause of death was postoperative bleeding in all patients (one patient was complicated with POPF). Pathologic curability (R0) was achieved in 145 patients (80.6%). Eighty-three patients (46.1%) had complications after pancreatectomy. Postoperative complications included DGE in 49 patients (27.2%), PF in 41 patients (22.8%), and surgical site infection in 13 patients (7.2%).

**Fig. 1:**
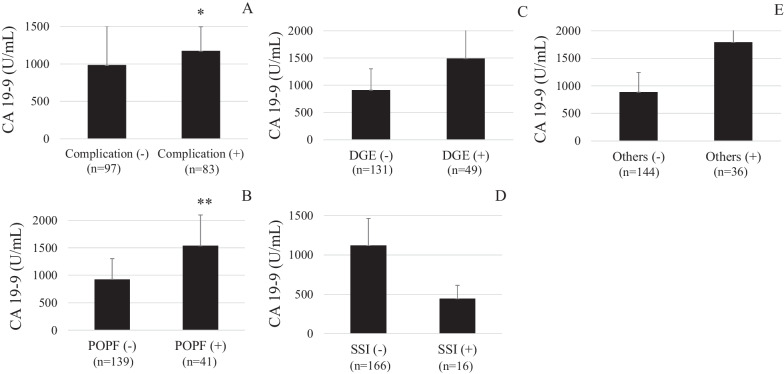
HYPERLINK "sps:id::fig1||locator::gr1||MediaObject::0" Preoperative serum CA19-9 levels in patients with various postoperative complications were compared with those of patients without various postoperative complications. A: any complication, B: postoperative pancreatic fistula (POPF), C: delayed gastric emptying, D: surgical site infection, E: others. *p < 0.05 vs. the patients without any postoperative complications; **p < 0.005 vs. the patients without POPF

Preoperative serum CA19-9 levels were significantly elevated in patients with postoperative complications than in patients without postoperative complications (Fig. 1). The receiver operating characteristic (ROC) curve resulted in a CA19-9 cutoff value of 398 U/mL and the area under the curve (AUC) of 0.596, predicting postoperative complications. The frequency of postoperative complications was also found to be significantly higher in patients with high CA19-9 compared to those with low levels (64.4% vs. 37.2%, P < 0.001).

**Fig. 2:**
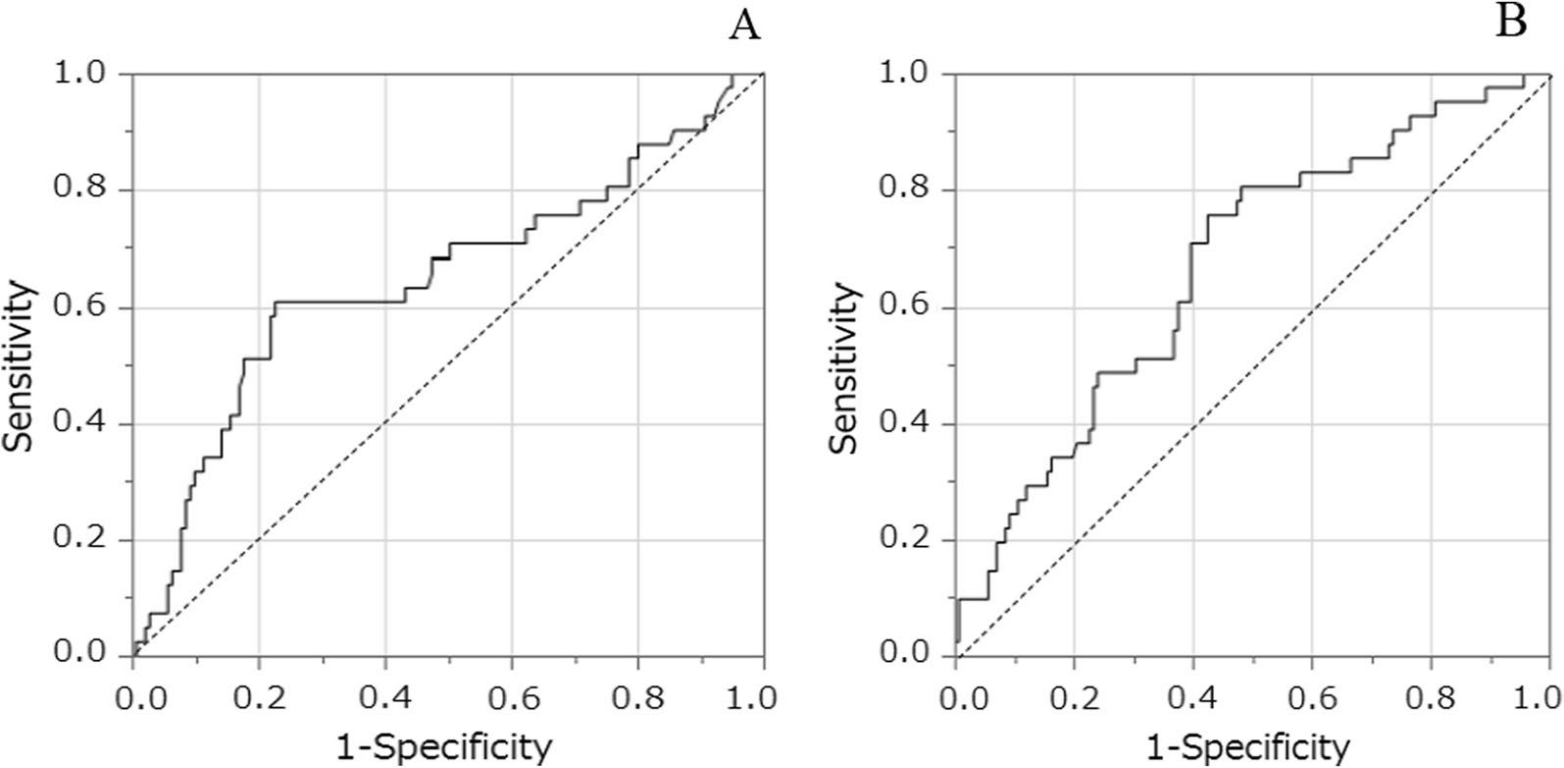
**A** The receiver operating characteristic (ROC) curve showed that the cutoff value of preoperative CA19-9 levels predicting POPF was 428 U/mL and the area under the curve (AUC) was 0.65 in patients undergoing pancreatectomy. **B** ROC curve showed that the cutoff BMI value predicting POPF was 21.7, and the AUC was 0.67 in patients undergoing pancreatectomy

CA19-9 levels were significantly higher in patients with POPF than in patients without POPF (P < 0.005) (Fig. 1). ROC curve resulted in a CA19-9 cutoff value of 428 U/mL and an AUC of 0.65, predicting POPF (Fig. 2A). Patients with high CA19-9 levels had a significantly higher frequency of POPF than those with low levels (43.9% vs. 13.0%, P < 0.0001) (Table 2).

**Table 2:** Risk factors for POPF in the cohort of 180 patients enrolled in this study

	Non-PF (n = 139)	PF (n = 41)	P-value
Gender (male/female)	76/63	20/21	0.44
Age	69.2 ± 0.8	67.9 ± 1.8	0.45
BMI	21.6 ± 0.3	23.6 ± 0.5	0.001
BMI ≥ 21.7	59 (42.4%)	31 (75.6%)	0.0001
ASA-PS			0.230
1	28 (20.1%)	12 (29.3%)	
≥ 2	111 (79.9%)	29 (70.7%)	
Preoperative biliary drainage	47 (33.8%)	12 (29.3%)	0.706
Histology: well-differentiated	61 (43.9%)	11 (26.8%)	0.046
Mean CA 19–9 (U/mL)	925 ± 380	1540 ± 556	0.0037
Median CA 19–9 (U/mL)	123	643	
CA19-9 ≥ 428 U/mL	32 (23.0%)	25 (61.0%)	< 0.0001
Tumor size (cm)	3.31 ± 0.13	3.41 ± 0.23	0.67
pT (UICC)			0.16
1	3 (2.2%)	2 (4.9%)	
2	3 (2.2%)	2 (4.9%)	
3	131 (94.2%)	34 (82.9%)	
4	2 (1.4%)	3 (7.3%)	
pN (UICC)			0.320
0	40 (28.8%)	17 (41.5%)	
1	63 (45.3%)	15 (36.6%)	
2	36 (25.9%)	9 (22.0%)	
pStage (UICC)			0.67
IA	3 (2.2%)	2 (4.9%)	
IB	2 (1.4%)	1 (2.4%)	
IIA	36 (25.9%)	14 (34.2%)	
IIB	62 (44.6%)	15 (36.6%)	
III	36 (25.9%)	9 (22.0%)	
Curability (R0)	117 (84.2%)	28 (68.3%)	0.03
Surgery			0.073
PD (n = 122)	99	23	
DP (n = 58)	40	18	
Operative time (min)	521 ± 41	492 ± 33	0.980
Bleeding (g)	1870 ± 133	2105 ± 298	0.50
Neoadjuvant chemotherapy	21 (15.1%)	7 (17.1%)	0.76

Other factors predicting POPF were investigated. BMI levels were significantly higher in the patients with POPF than in those without POPF (P = 0.001). ROC curve resulted in a BMI cutoff value of 21.7 and AUC of 0.67, predicting POPF (Fig. 2B). The frequency of POPF was also found to be significantly higher in patients with higher BMI values than in those with lower values (34.4% vs. 11.1%, P = 0.0001) (Table 2). The frequency of POPF was also significantly higher in patients with not well differentiated adenocarcinoma than in patients with well-differentiated adenocarcinoma (P = 0.046) (Table 2). We also found that CA19-9 was significantly higher in patients with not well differentiated adenocarcinomas than in those with well differentiated adenocarcinomas (P < 0.01) (Table 3). The frequency of POPF in patients with R1 or R2 was also significantly higher than in patients with R0 (P < 0.05) (Table 2). CA19-9 levels in R1 or R2 patients were significantly higher than in R0 patients (P < 0.05) (Table 3).

**Table 3:** Relationship between CA19-9 levels and histology or curability

	CA19-9 (U/mL)	CA19-9 ≥ 428 (n = 57)
Histology		
Not well-differentiated (n = 108)	1431 ± 520	42 (38.9%)
Well-differentiated (n = 72)	517 ± 158	15 (20.8%)
P-value	P < 0.01	*P* < 0.005
Curability		
R0 (n = 145)	496 ± 87	42 (29.0%)
R1 or R2 (n = 35)	3456 ± 1558	15 (42.9%)
P-value	P = 0.084	*P* < 0.05

However, age, sex, ASA-PS, preoperative biliary drainage, tumor size, T-factor, lymph node metastases, surgical method, pathological stage, surgical blood loss, operative time and neoadjuvant chemotherapy were not associated with POPF (Table 2). There was no difference of the incidence of POPF between pancreatic anastomosis procedures.

Multivariate analysis demonstrated that preoperative serum CA19-9 ≧428 U/mL (odds ratio [OR]: 4.36, 95%, confidence interval [CI]: 1.96–9.68) and BMI ≧21.7 (OR: 4.06, CI: 1.76–9.37) were statistically significant independent risk factors for POPF (Table 4).

**Table 4:** Multivariate analysis of risk factors for POPF

Risk factor	Odds ratio	95% CI		P-value
CA19-9 ≥ 428 U/mL	4.36	1.96	9.68	0.0003
BMI ≥ 21.7	4.06	1.76	9.37	0.001
Curability (R0)	1.99	0.79	5	0.145
Histology (well-differentiated)	0.66	0.28	1.53	0.33

We investigated whether the significance of CA19-9 levels for POPF differs by operative method. In 122 patients who underwent pancreaticoduodenectomy, CA19-9 levels were significantly higher in patients with POPF than in those without, and patients with higher CA19-9 levels had a significantly higher incidence of POPF than those with lower levels (35.9% vs. 10.8%, P < 0.005) (Table 5). In 122 patients who underwent pancreaticoduodenectomy, 58 patients underwent preoperative biliary drainage. Preoperative biliary drainages included endoscopic biliary drainage (EBD) in 50 patients, percutaneous transhepatic biliary drainage (PTBD) in 5 patients and percutaneous transhepatic gallbladder drainage (PTGBD) in 3 patients. Serum CA19-9 levels were higher in patients with preoperative biliary drainage than in those without. However, preoperative biliary drainage was not correlated with POPF. Preoperative total bilirubin levels were not corelated with POPF, although they were corelated with CA19-9 levels. On the other hand, size of Wirsung duct were not correlated with POPF or CA19-9 levels (Additional file 1). In 58 patients who underwent distal pancreatectomy, CA19-9 levels were higher in patients with POPF than in patients without POPF, and the frequency of POPF was significantly higher in patients with high CA19-9 levels than in patients with low CA19-9 levels (61.1% vs. 17.5%, P < 0.005) (Table 5).

**Table 5:** Relationship between CA19-9 and POPF by operative method

	CA19-9 (U/mL)	CA19-9 < 428 (n = 123)	CA19-9 ≥ 428 (n = 57)
PD (n = 122)		83	39
Non-PF (n = 99)	1109 ± 523	74 (89.2%)	25 (64.1%)
PF (n = 23)	1808 ± 917	9 (10.8%)	14 (35.9%)
P-value	*P* = *0.024*	*P* = *0.0014*	
DP (n = 58)		40	18
Non-PF (n = 40)	469 ± 255	33 (82.5%)	7 (38.9%)
PF (n = 18)	1199 ± 503	7 (17.5%)	11 (61.1%)
P-value	P = 0.054	*P* = *0.0011*	

### Postoperative course in the patients who underwent pancreatectomy

Length of hospital stay for patients with POPF was significantly longer than for patients without POPF (P < 0.0001). Patients with elevated CA19-9 had longer hospital stays than those with low levels (P = 0.07) (Table 6). A total of 147 (81.7%) patients underwent adjuvant chemotherapy. Patients with POPF received postoperative adjuvant chemotherapy less frequently than patients without POPF (73.2% vs. 84.2%, not significantly, P = 0.12).

**Table 6:** Short- and long-term outcomes

	CA19-9 < 428 (n = 123)	CA19-9 ≥ 428 (n = 57)	P-value
Postoperative complications	47 (38.2%)	36 (63.2%)	< 0.005
POPF	16 (13.0%)	25 (43.9%)	< 0.0001
Hospital stay (days)	40.6 ± 2.1	44.7 ± 3.0	0.07
In-hospital mortality	3	0	
Adjuvant chemotherapy	101 (82.1%)	46 (80.7%)	0.82
Median DFS (m)	23	8	< 0.0001
5-year DFS	36.50%	13%	
Median OS (m)	56	18	< 0.0001
5-year OS	43.40%	15.40%	

### Long-term outcomes of pancreatectomy for PDAC

**Fig. 3:**
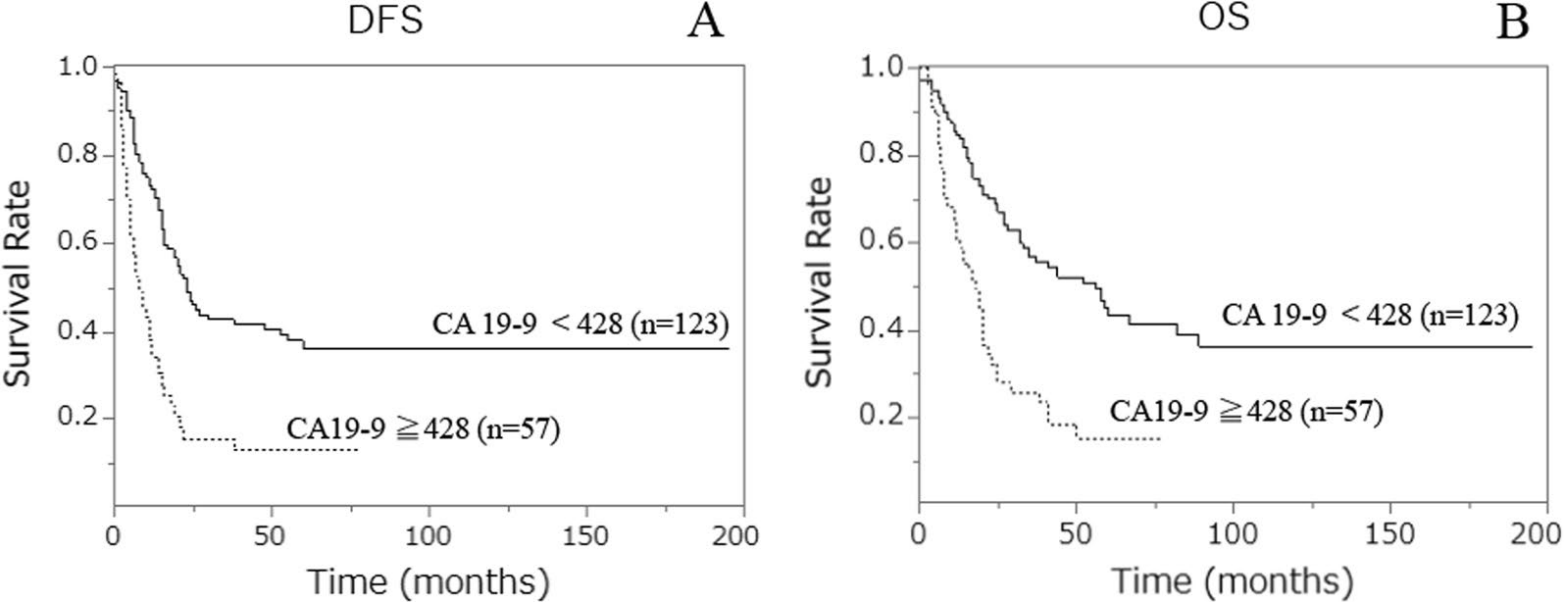
**A** Disease-free survival curves for high and low preoperative serum CA 19-9 levels in patients undergoing pancreatectomy for pancreatic ductal adenocarcinoma (PDAC). Significant differences in survival rates were observed between patients with high and low CA19-9 levels (log-rank test, P < 0.0001). **B** Overall survival curves for high and low preoperative serum CA19-9 levels in patients undergoing pancreatectomy for PDAC. Significant differences in survival rates were observed between patients with high and low CA19-9 levels (log-rank test, P < 0.0001)

Figure 3 shows Kaplan–Meier survival curves by CA19-9 level in patients who underwent pancreatectomy. The median observation period was 32 months. Patients with higher CA19-9 levels had significantly shorter DFS and OS than those with lower CA19-9 levels (P < 0.0001). Table 6 shows a summary of short- and long-term outcomes after pancreatectomy by CA19-9 levels.

## Discussion

We investigated whether elevation of preoperative CA19-9 levels is associated with short- and long-term outcomes after pancreatectomy for PDAC. Multivariate analysis shows that high levels of CA19-9 and BMI were statistically significant independent risk factors for PF after pancreatectomy. DFS and OS of patients with higher levels of CA19-9 were significantly shorter than those with lower levels of preoperative serum CA19-9.

CA19-9 is a tumor marker which is known to be a Sialyl Lewis-a antigen. This antigen is normally present in salivary mucus and in exocrine pancreatic secretions. Elevated serum CA19-9 levels are found in 69–92% of patients with pancreatic cancer [14]. CA19-9 is not detectable in the patients with Le^a−b−^ phenotype. Approximately 5% of the population belong to this group. Thus, 16 patients (8.1%), whose CA19-9 were not detected, were eliminated in analysis.

Elevation of CA19-9 levels is useful for diagnosis of pancreatic cancer [14]. Also, previous reports have demonstrated that elevation of CA19-9 levels is a long-term prognostic factor [20]. CA19-9 is produced in pancreatic cancers and expressed on cancer cell surfaces, induces cancer cells to attach to vascular endothelial cells by E-selectin and results in metastasis.

This is the first paper reporting significance of higher preoperative serum CA19-9 as an independent risk factor for PF in patients who undergo pancreatectomy for PDAC. Sufficient investigation of the relationship between CA19-9 levels and POPF has not been reported, because most of papers had investigated the risk factors in cohorts including the patients who had pancreatic diseases other than PDAC. We can formulate some hypothesis to why elevation of CA19-9 levels is an independent risk factor for PF after pancreatectomy. CA19-9 levels are elevated in the patients with advanced and aggressive cancer generally, in which surgical stress is greater. Therefore, the patients experienced hypercatabolic conditions after surgery compared to the patients with not-elevated CA19-9 levels. This study demonstrated that curability and histology were associated with CA19-9, and both of them were associated with POPF. These results support this hypothesis. However, this hypothesis conflicts with the results that cancer stage, bleeding or operative time were not related to PF after pancreatectomy in this study. Main pancreatic duct (MPD) stenosis or obstruction results in elevation of serum CA19-9 levels [21]. High serum CA19-9 levels may be one of the results of pancreatitis that causes delayed pancreatic tissue repair [22]. Serum CA19-9 elevation in chronic pancreatitis is not so high compared to pancreatic cancer related CA19-9 elevation. Serum CA19-9 elevation is associated with cancer aggressiveness and may be associated with tumor growing speed. Previous papers reported that POPF was associated with hard pancreatic texture contributes to suppression of pancreatic fistula development. Hard of pancreatic tissue means fibrosis of the pancreatic tissue. In patients with higher CA19-9 levels, fibrosis of pancreatic tissue has not yet occurred. because there may not be enough time for inflammation and fibrosis to development. In addition, CA19-9 may be a biological marker of occult metastases, because CA19-9 was a significant long-term prognostic factor. Metabolic changes resulting from metastatic cancer may affect complications and tissue repair after pancreatectomy, although the mechanism is unknown. These may be the reason why pancreatic fistulas are more likely to occur in patients with higher CA19-9. In this study, CA19-9 was associated with histology. The results support this hypothesis. Note that the texture of pancreatic tissue was not investigated in this study because an appropriate method for measuring the hardness of pancreatic tissue has not been established.

BMI was an independent risk factor for POPF in this study. These results are consistent with previous reports [23, 24]. Higher CA19-9 levels is an independent risk factor. On the other hand, curability and histology were significant risk factors in univariate analysis, but not independent risk factors in multivariate analysis. The reason for this may be that the curability and histology correlated with CA19-9 levels.

POPF is a critical complication of pancreatectomy and sometimes induces arterial wall breakdown followed by major bleeding into the peritoneal cavity. Therefore, it is important to know the risk of POPF before surgery. Recent papers reported that DFS and OS of the patients with high levels of CA19-9 were shorter compared to the patients with low levels [15, 16]. Higher CA19-9 was a significant long-term prognostic factor in this study. CA19-9 was produced by pancreatic cancer cells and may be associated with aggressiveness of the tumor. On the other hand, POPF may be involved in poor long-term prognosis, because the rate of patients with post-operative adjuvant chemotherapy was lower in the patients with POPF compared to the patients without POPF (P = 0.12). The importance of neoadjuvant chemotherapy and decreased CA19-9 levels before surgery for improvement of the long-term prognosis had been reported [25, 26].Therefore, preoperative CA19-9 may be important for the management of PDAC. This is retrospective analysis at a single institution, therefore large-scale prospective cohort studies are necessary in the future.

## Conclusion

Preoperative serum CA19-9 levels are a significant predictor of POPF in PDAC patients undergoing pancreatectomy.

## Supplementary Information

**Additional file 1: Table S1.** Relationship between CA19-9 and the risk factors in patients who underwent PD. **Table S2.** Relationship between pancreatic fistula and risk factors in patients who underwent PD

**Additional file 2: **Analysis of 196 PDAC patients

## Data Availability

All data generated or analyzed during this study are included in this published article [and its Additional files 1 and 2].

## Abbreviations

CA 19-9: Carbohydrate antigen 19-9
PDAC: Pancreatic ductal adenocarcinoma
POPF: Postoperative pancreatic fistula
PD: Pancreaticoduodenectomy
DP: Distal pancreatectomy
DGE: Delayed gastric emptying
ROC curve: Receiver operating characteristic curve
DFS: Disease free survival
OS: Overall survival
