# Supplementary material for: Preoperative serum CA19-9 predicts postoperative pancreatic fistula in PDAC patients: retrospective analysis at a single institution
Source: BMC Surg. 2022 Oct 28;22:367. doi: 10.1186/s12893-022-01825-3 (PMC9617438; doi:10.1186/s12893-022-01825-3)
Supplement: Supplementary file 1 — Additional file 1: Table S1. Relationship between CA19-9 and the risk factors in patients who underwent PD. Table S2. Relationship between pancreatic fistula and risk factors in patients who underwent PD [file 12893_2022_1825_MOESM1_ESM.docx]

**Additional file 1**

**Table S1**  Relationship between CA19-9 and the risk factors in patients who underwent PD

| PD (n=122) | median CA19-9 (U/mL) | CA19-9 < 428 (n=83) | CA19-9 ≥ 428 (n=39) | P-value |
| --- | --- | --- | --- | --- |
| Biliary drainage |  |  |  |  |
| no (n=64) | 88.1 | 49 (59.0%) | 15 (38.5%) |  |
| yes (n=58) | 237.9 | 34 (28.9%) | 24 (61.5%) | P=0.051 |
| P-value | *P<0.01* |  |  |  |
| Total bilirubin |  |  |  |  |
| median |  | 0.7 | 1.2 | *0.032* |
| mean $\boldsymbol{\pm}$ SD |  | 1.29 $\boldsymbol{\pm}$ 1.52 | 1.84 $\boldsymbol{\pm}$ 1.83 | 0.087 |
| Size of Wirsung duct |  |  |  |  |
| median |  | *4.5* | *5.0* | *0.59* |
| mean $\boldsymbol{\pm}$ SD |  | *4.98* $\boldsymbol{\pm}$ *2.12* | *5.35* $\boldsymbol{\pm}$ *2.53* | 0.40 |

**Table S2**  Relationship between pancreatic fistula and risk factors in patients who underwent PD

| PD (n=122) | non-PF (n=99) | PF (n=23) | P-value |
| --- | --- | --- | --- |
| Biliary drainage |  |  |  |
| no (n=64) | 52 (52.5%) | 12 (52.2%) |  |
| yes (n=58) | 47 (47.5%) | 11 (47.8%) | P=1.0 |
| Total bilirubin |  |  |  |
| median | 0.8 | 0.9 | 0.72 |
| mean $\boldsymbol{\pm}$ SD | 1.37 $\boldsymbol{\pm}$ 1.35 | 1.90 $\boldsymbol{\pm}$ 2.52 | 0.16 |
| Size of Wirsung duct |  |  |  |
| median | 5.0 | 5.0 | 0.59 |
| mean$\boldsymbol{\pm}$SD | 5.13 $\boldsymbol{\pm}$ 2.21 | 4.97 $\boldsymbol{\pm}$ 2.47 | 0.76 |
