# Supplementary material for: Preoperative serum CA19-9 predicts postoperative pancreatic fistula in PDAC patients: retrospective analysis at a single institution
Source: BMC Surg. 2022 Oct 28;22:367. doi: 10.1186/s12893-022-01825-3 (PMC9617438; doi:10.1186/s12893-022-01825-3)
Supplement: Supplementary file 2 — Additional file 2: Analysis of 196 PDAC patients [file 12893_2022_1825_MOESM2_ESM.docx]

**Analysis of 196 PDAC patients**

Among one hundred ninety-six patients undergoing pancreatectomy for PDAC, 47 patients were complicated with POPF. Median preoperative CA19-9 levels in patients with PF (n=47) and without PF (n=149) were 459.0 and 100.7 U/mL, respectively (P=0.057). The ROC curve showed that the cutoff CA19-9 value predicting POPF was 428 U/mL, and the AUC was 0.592. The frequency of POPF was also significantly higher in the patients with high CA19-9 levels than in those with low CA19-9 levels (43.1% vs. 15.9 %, P<0.0001).
